# Designing mHealth for maternity services in primary health facilities in a low-income setting – lessons from a partially successful implementation

**DOI:** 10.1186/s12911-018-0704-9

**Published:** 2018-11-12

**Authors:** Solomon Shiferaw, Andualem Workneh, Robel Yirgu, Geert-Jan Dinant, Mark Spigt

**Affiliations:** 1 Department of Reproductive Health and Health Service Management, School of Public Health, College of Health Sciences, Addis Ababa University, Addis Ababa, Ethiopia; 2 Individual Consultant for the project, Addis Ababa, Ethiopia; 3 CAPHRI School for Public Health and Primary Care, Department of Family Medicine, Maastricht University, Maastricht, The Netherlands; 4 General Practice Research Unit, Department of Community Medicine, the Arctic University of Norway, Tromsø, Norway

**Keywords:** mHealth, Antenatal and postnatal care, Maternal health, Open data kit (ODK)

## Abstract

**Background:**

Increasing mobile phone ownership, functionality and access to mobile-broad band internet services has triggered growing interest to harness the potential of mobile phone technology to improve health services in low-income settings. The present project aimed at designing an mHealth system that assists midlevel health workers to provide better maternal health care services by automating the data collection and decision-making process. This paper describes the development process and technical aspects of the system considered critical for possible replication. It also highlights key lessons learned and challenges during implementation.

**Methods:**

The mHealth system had front-end and back-end components. The front-end component was implemented as a mobile based application while the back-end component was implemented as a web-based application that ran on a central server for data aggregation and report generation. The current mHealth system had four applications; namely, data collection/reporting, electronic health records, decision support, and provider education along the continuum of care including antenatal, delivery and postnatal care. The system was pilot-tested and deployed in selected health centers of North Shewa Zone, Amhara region, Ethiopia.

**Results:**

The system was used in 5 health centers since Jan 2014 and later expanded to additional 10 health centers in June 2016 with a total of 5927 electronic forms submitted to the back-end system. The submissions through the mHealth system were slightly lower compared to the actual number of clients who visited those facilities as verified by record reviews. Regarding timeliness, only 11% of the electronic forms were submitted on the day of the client visit, while an additional 17% of the forms were submitted within 10 days of clients’ visit. On average forms were submitted 39 days after the day of clients visit with a range of 0 to 150 days.

**Conclusions:**

In conclusion, the study illustrated that an effective mHealth intervention can be developed using an open source platform and local resources. The system impacted key health outcomes and contributed to timely and complete data submission. Lessons learned through the process including success factors and challenges are discussed.

## Background

Mobile based health management information systems have multiple advantages over the paper based systems including among others, reducing human error, eliminating the need for double data entry, easing the process of communicating data from remote rural communities to central locations, and obtaining real time data with low cost [1–3]. Fueled by the increasing mobile phone ownership, functionality and access to mobile-broad band internet services, there is a growing interest to harness the potential of mobile phone technology to improve health services in lowincome settings [4]. Previous studies have shown the positive impact of mHealth applications in improving maternal health outcomes in low-income settings [5–8]. Key areas of health system improvements include adherence, appointment compliance, data collection, point of care service, health promotion and developing support networks for health workers [6, 7].

However, there is limited evidence on the effectiveness of mHealth interventions beyond the specific research projects [7, 9]. Much too often the mHealth application is no longer used when the (usually very expensive) project stops. Well-known challenges to scale-up or even maintain existing mHealth interventions include lack of sustainable financing [10, 11], as well as weak organizational structure and culture to fully adopt the Information Communication Technology (ICT) infrastructure. Lack of capacity to adapt technology to low-income settings, complexity of ensuring interoperability and integration of information systems and securing privacy of information also remain important technical challenges [11].

Additional concerns are questions about cost-effectiveness of mHealth interventions, especially for non-open source applications, which might involve significant initial investment as well as maintenance cost and the questionable role of industry’s increasing involvement in pushing for mHealth scale-up [12]. A systematic literature review on the cost-utility and cost-effectiveness of mHealth systems and other eHealth interventions by Isabel et al., [13] showed that there is paucity of evidence in this regard because of few randomized controlled trials, small sample sizes, and the absence of quality data although there are studies currently underway to document cost-effectiveness [14, 15].

Currently we lack understanding of implementation challenges and what it takes to develop a locally viable project that could last beyond the projects’ limited timeline [5, 16]. It is in light of these global and national developments that the current project, which aimed at exploring the potential use of mobile phone-based applications to impact maternity service utilization in primary care facilities, was conceived and implemented in Ethiopia. The actual effect of the mHealth intervention on improving maternity service utilization (specifically antenatal care and institutional delivery) has been published elsewhere [17]. In summary the study showed that an mHealth application can improve adherence to repeat ANC visits, delivery care and early postnatal care in a low-resource environment using controlled prospective evaluation of the intervention over a period of 12 months. The present paper attempted to fill as much of this gap as possible, by suggesting key lessons learned in the process of designing mHealth interventions in a poorly resourced setting, so that future initiatives can have a better chance of success.

**Fig. 1:**
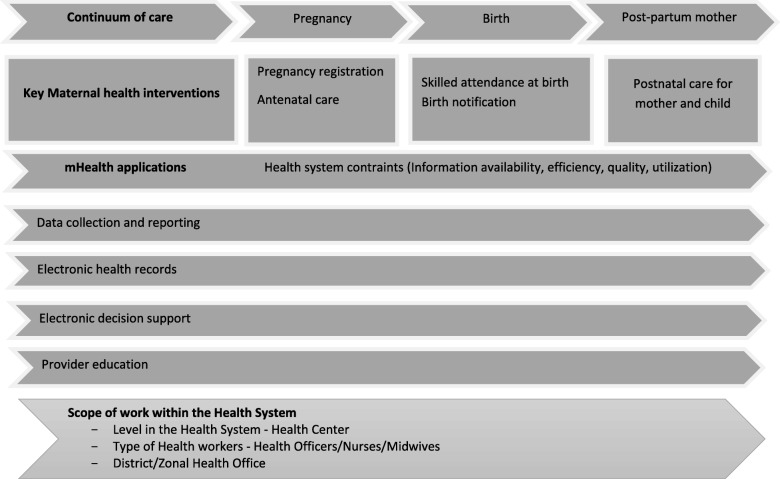
The mHealth and ICT framework

In this paper, we describe the processes involved in designing the mHealth application using a modified version of the health systems framework suggested by Alain B et al. as shown in Fig. 1 [14]. Also described are details of implementation including training local IT professionals and the intervention’s possible contributions in advancing data collection, aggregation, reporting and analysis, enhancing decision support, strengthening feedback and promoting the culture of information use in the process. Challenges encountered during implementation are highlighted with potential solutions for future initiatives.

### The problem

The region where the current intervention was implemented had relatively low rates of antenatal and delivery service utilization, which made it a priority for local health officials and eventually helped in having a great sense of local ownership. The following table (Table 1) summarizes key maternity service utilization indicators in contrast to the national average using the most recent national survey at the time – Ethiopian Demographic and Health Survey 2011 [18].

**Table 1:** Percent distribution of women age 15–49 who had a live birth in the five years preceding the survey by selected maternity services for the most recent birth, 2011

Indicator	Amhara (%)	National (%)
Antenatal Care (ANC) from a skilled provider^a^	33.6	33.9
Specific ANC services^b^		
Informed of signs of pregnancy complications	16.4	20.0
Blood pressure measured	61.1	71.6
Urine sample taken	37.4	41.3
Delivered by a skilled attendant^c^	10.1	10.0
Postnatal check-up in the first 2 days after birth^d^	5.1	6.7

^a^Percentage women age 15–49 receiving antenatal care from a skilled provider for the most recent birth

^b^Among women receiving antenatal care (ANC) for the most recent live birth in the 5 years preceding the survey, the percentage receiving specific antenatal services

^c^Percent distribution of births in the 5 years preceding the survey delivered by skilled providers

^d^Percentage of women with a live birth in the 2 years preceding the survey who received a postnatal checkup in the first 2 days after giving birth

## Methods

### Setting

The study was conducted in North Shewa Zone, Amhara region, which is located 130 Kilometers North East of the capital Addis Ababa. Five intervention health centers, each serving an average of 25,000 people, were involved in the study. Health centers are primary health care facilities staffed with midlevel health workers. All intervention health centers were within 10 Kilometers from the main road from Addis Ababa going to North eastern direction, to ensure they have comparable access to mobile phone network.

The health service system in Ethiopia is federally decentralized along the nine regions and two administrative city councils. Each of the nine regions is divided into Zones and each Zone into lower administrative units called Woredas, or Districts. Each Woreda is subdivided into the lowest administrative unit, called a Kebele. The health system is organized in three tiers as primary, secondary (General hospital) and tertiary (Specialized hospital). The primary health care level includes a District hospital (which cater for up to 100,000 people) along with a health center and 5 satellite health posts which together serve on average 25,000 people [19]. The health centers are also staffed with Health Information Technicians who are charged with the responsibility of improving the computer skills of the staff in the unit, report health data upwards in the system and extract health data for local use to improve the quality of care [20].

#### Training and capacity building

A two-days training was given to 15 health care professionals (3 from each health center) which was repeated every 3 months (2 days each) to refresh their memory and get feedback on ongoing challenges. To ensure that the system would continue to run after the initial pilot period, the project team additionally trained three members of the Zonal Health bureau IT professionals, and two health officials on the basics of the application including designing new forms and setting-up local servers, if needed. Additionally, the team provided two servers and 15 phones as back-up for future use.

### Ethical considerations

The original research was approved by the Institutional Review Board (IRB) of College of Health Sciences at Addis Ababa University (Protocol number 040/12/SPH) and findings of the controlled intervention study was published on PLOSONE – available at DOI:10.1371/journal.pone.0158600. Verbal consent was obtained from participants (clients of Antenatal, Delivery or Postnatal Care) after information about the study was given as required by the local IRB. Participants were informed that their participation is voluntary, their information will remain anonymous and that they are free to withdraw from the study at any point in time. The IRB approved verbal consent procedures (without a need for written consent) as it is customary for simple questionnaire surveys without any invasive procedures in an environment where literacy is relatively low. Like other surveys, women 15–17 were considered as emancipatory minors capable of giving consent to the study as per the national Research Ethics Review Guideline – available at http://www.ccghr.ca/wp-content/uploads/2013/11/national-research-ethics-review-guidline.pdf. The datasets used and/or analysed during the current study are available from the corresponding author on reasonable request.

### Use case description and system users

The system had front-end and back-end components. The front-end component was implemented as mobile phone-based application that was used by health workers. The back-end component was implemented as a web-based application that ran on a central server for data aggregation and report generation. The user groups interacted with the system through the front-end (mobile phone-based) or back-end (computer-based) applications (Table 2).

**Table 2:** User groups who interacted with the mHealth system

User Groups	Description
Health care providers	Health workers (health officers^a^ and nurses/midwives) who provided maternity care service (i.e. antenatal, delivery and postnatal care). These users interacted with the system through a mobile phone application.
Supervisors at District/Zonal health office	Health service administrators and supervisors to whom health care providers, at health centers, were supposed to report. Users under this group interacted with the system through a back-end web-based application to retrieve the data and generate a more detailed analysis.
System Administrator	System Administrator was an IT professional that indirectly interacted with the system to do system maintenance, administration, and database backup activities.

^a^Health Officers – are mid-level professionals who receive 4 years of clinical and public health training

**Fig. 2:**
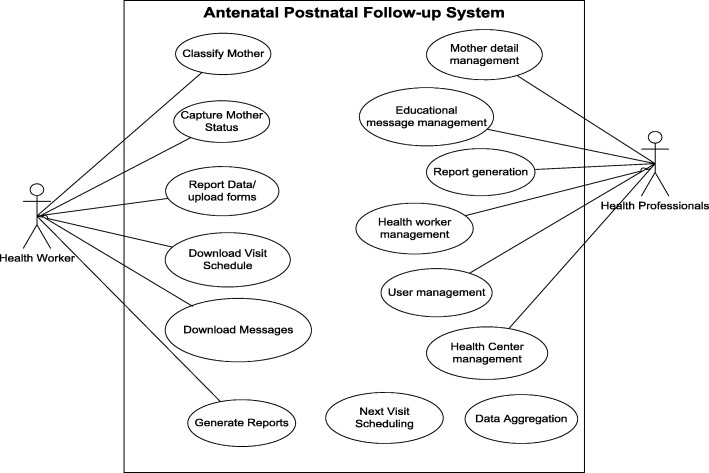
Use case diagram

Health workers interacted with different features of the mHealth system through the Antenatal Care-Postnatal Care (ANC-PNC) mobile based application (Fig. 2). However, two of the system’s features, “Next Visit Scheduling” and “Data Aggregation” did not require user intervention. Rather they were executed based on the system’s internal triggers and conditions.

### System development approach and tools

The technical requirements of the system were determined by an IT expert hired for this purpose. The principal investigator provided relevant documents including current data collection forms in health centers, schedule for ANC visits and Expected Data of Delivery (EDD) calculation logics. The IT expert used these documents and additional resources (journal and online articles) to develop the first version of the system as a prototype. Internal system-level testing and integration-testing was conducted by the IT expert to identify and fix issues. The process of development and feedback gathering was repeated iteratively until the system became good enough to move to end users.

A similar feedback scheme was used with end users to iteratively update the system based on their day to day work experience. During the first field visit and user training sessions, the system’s functionalities and features were presented to health workers with the intention of introducing the system and gathering more feedback. Content of the electronic forms were reviewed with users to identify missing questions and issues in question sequencing and wording. Iterations of development and feedback gathering were conducted with health workers during subsequent training sessions where users participated in testing the system before it was deployed in a production environment for piloting.

Bearing in mind the existing limited infrastructure at health centers, the system was designed and developed by considering the constraints listed out in Table 3 below.

**Table 3:** Solution constraints and their rationale

Solution Constraint	Rationale
The front-end application ran on a mobile phone	It is not feasible and affordable to use other computing devices such as personal computers.
The front-end application had to work in offline mode	There is limited network connectivity in the selected target environment.
The system used open-source technologies	This constraint was required to minimize the development costs of the system.
The system should also be publicly available as an open-source product	The system is not developed for profit. Rather it is to be used by the regional health Office as well as other interested parties.

### Off-the-shelf software

To fulfill part of the system’s requirement, an open source data collection tool called Open Data Kit (ODK) was customized [21]. The term “open source software” refers to a software that people can use, modify and share because its design is publicly accessible [22]. ODK has three major tools called ODK Build, ODK Collect, and ODK Aggregate. ODK Build is a web-based cloud application that is used to develop electronic forms for mobile data collection. ODK Collect helps users to collect and upload data using electronic forms. ODK Aggregate is a ready-to-deploy web-based server application used as a data repository. It has data visualization and report generation features and provides a means to receive filled forms from ODK Collect and manage collected data.

For the current mHealth system, all the three software tools were used to implement part of the system’s requirement. ODK Build was used to develop five electronic forms that were used to collect mother’s health status during antenatal, delivery, and postnatal care visits. In addition, two additional electronic forms were also developed with ODK Build for the baseline and end-line exit surveys among antenatal care clients. ODK Collect was customized to include the following features in order to fulfill the system’s requirement;

- Remind health workers about pregnant woman’s next visit date;
- Link related forms to enable data integration during the longitudinal follow-up;
- Download visit schedule, recent visit detail, and educational messages;
- Generate reports about mother’s visit for any given time period;

ODK Aggregate was used for data aggregation at the central server. Additional features that were required from the system were developed as a separate web-based application and interfaced with ODK Aggregate at database level. The newly developed features that were implemented in the web platform developed for the purpose included:

- Pregnant women’s data management (which has details about number of women with each scheduled ANC visits, delivery, PNC and other related health conditions);
- Educational message management (on complaints during pregnancy and danger signs during pregnancy);
- Health worker and health center management (which allows disaggregation by health center and individual health worker); and
- Next visit scheduling module (information about the scheduled 4 ANC visits for each woman);

### Implementation environment and system design

**Fig. 3:**
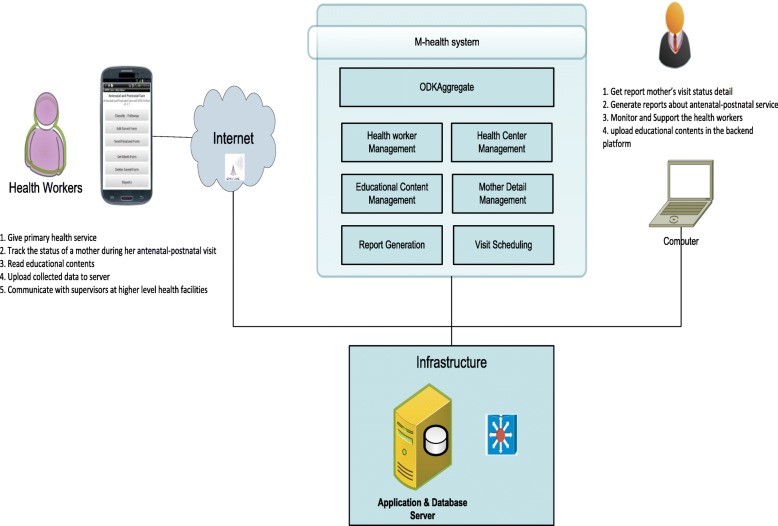
High level System Architecture

The system had a client-server architecture that used mobile phones at the client side and a web-based application on the server side. At the client side, health workers could use the mobile application to interact with the server system at Addis Ababa University server center. The interaction between the client and the server systems was through Ethio-Telecom’s GPRS connectivity. The system’s high-level architecture is shown in Fig. 3.

The system was designed to work in an offline mode, so that collected data could be saved in the mobile phone’s internal memory until network connectivity was available. A mobile phone model with longer battery life was selected to run the front-end application for longer periods of time without requiring frequent recharging. At the back-end platform, health professionals and/or supervisors working at the Zonal Health Office could interact with the mHealth system using their personal computers. It is worth noting that health officials were able to monitor the activities of health workers from the back-end application, which helped them to make timely decisions based on reports submitted by health workers.

### Mobile application

**Fig. 4:**
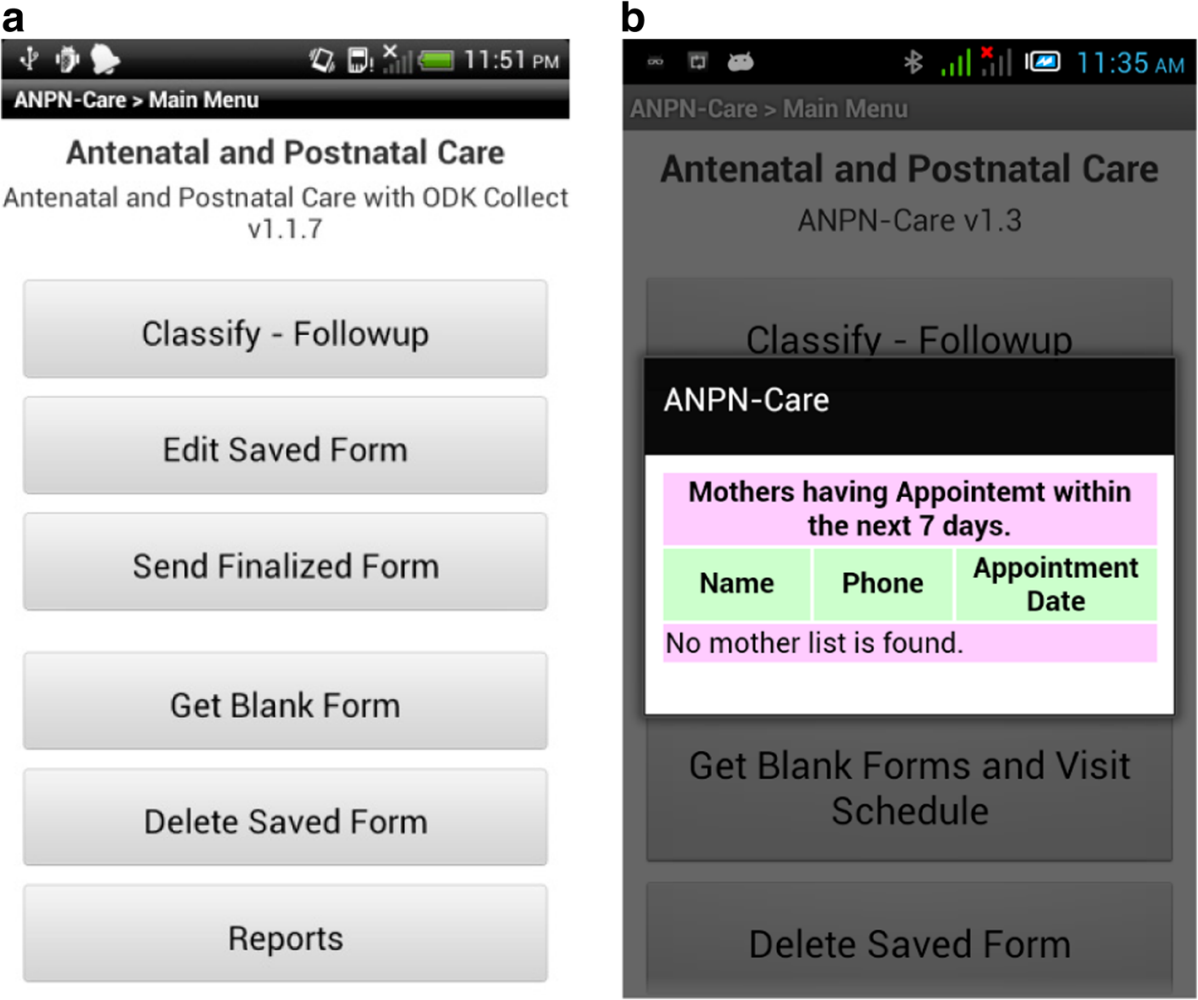
**a** Appointment reminder; **b** Mobile application’s main menu

The front-end application was developed as an android-based mobile application. An android operating system was chosen because of its capability to be localized and customized easily. The front application’s main menu is shown in Fig. 4a. Whenever there was network connectivity, the form could be sent to the main server by using “Send Finalized Form” option (Fig. 4a). Whenever the application was launched, it automatically displayed a reminder about list of pregnant women who had a scheduled visit for the following 7 days. Pregnant women’s next visit schedule was computed by the back-end application, so that health workers could get the schedule from their front-end mobile application. The visit schedule reminder dialog box that was presented to health workers is shown in Fig. 4b. Once the form was filled and finalized, the collected data was saved in the mobile phone’s memory.

**Fig. 5:**
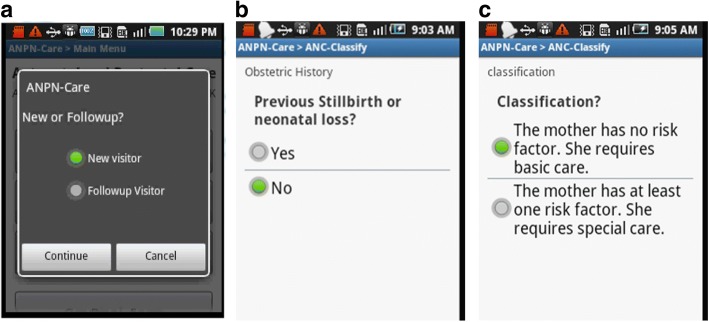
**a** New or follow-up visitor; **b** Example of questions used to classify women; **c** Suggested classification by the system

When a new visitor came for antenatal care, the health worker was expected to use the first form labeled as, “Classify-Follow up”. As shown in Fig. 5a, the system asked whether the visit was made for the first time or whether it was a follow-up visit. Based on the user’s response, an appropriate form was opened. As shown in the sample case, a classification form, “ANC-Classify” was opened by the application (Fig. 5b) to collect the pregnant woman’s health status and to decide whether or not she required basic or specialized care (Fig. 5c). This classification was made by the system itself based on the pregnant woman’s previous and current medical and obstetric history.

**Fig. 6:**
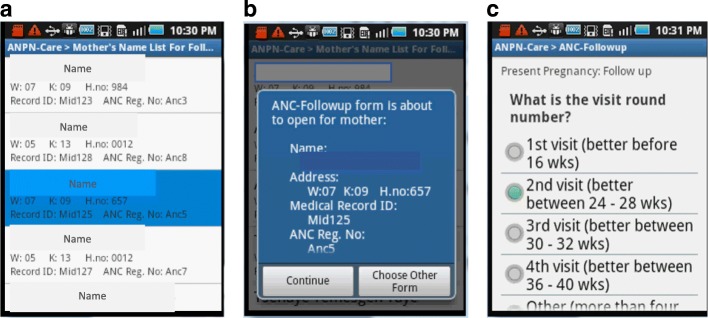
**a** List of pregnant women expected for follow-up visit, **b** Proposed current visit form for selected woman, **c** Pregnancy Follow-up form for selected woman

If the woman came for a follow-up visit, her previous visit status could be downloaded from the back-end system. This downloaded list of visit status contains a list of pregnant women who are expected for a follow-up visit (Fig. 6a). Then based on the woman’s last visit status, an appropriate form was proposed by the system for the current visit (Fig. 6b), so that the health worker could fill-in relevant data about her current health status and report the data to the back-end system (Fig. 6c). Note that names shown below are random names used for demonstration/training purposes and do not refer to actual women who participated in the study.

**Fig. 7:**
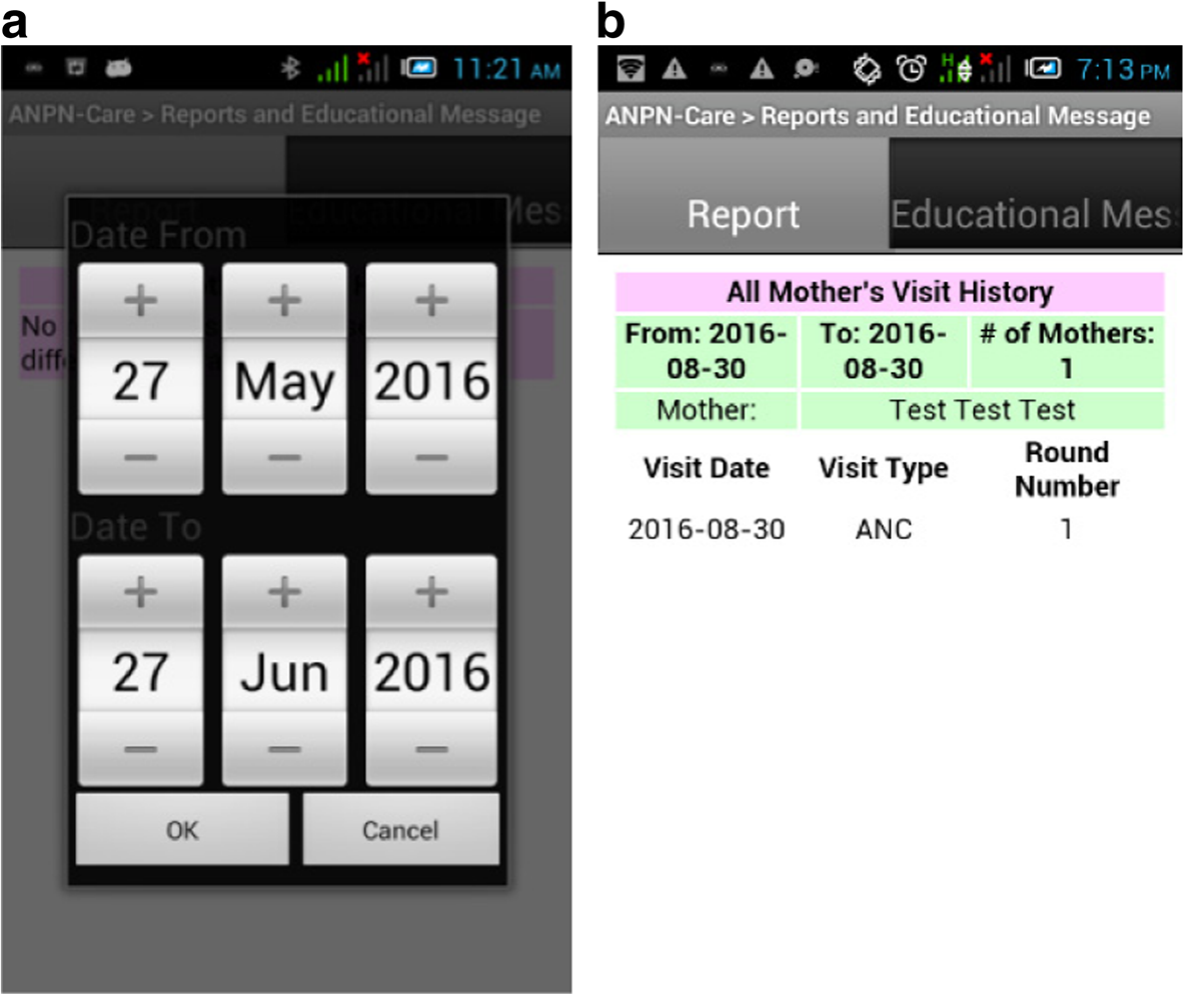
**a** User Interface to select date range for report. **b** List of pregnant women who visited during a given date range (visit date, type of visit and round of visit)

Health workers could also generate reports from their front-end application. In order to do so, the front-end application interacted with the back-end system to get the pregnant woman’s visit report for a given date range. This feature helped the health worker to easily compile what s/he had accomplished during a given period. Figure 7a and b shows report generating feature of the mobile application.

**Fig. 8:**
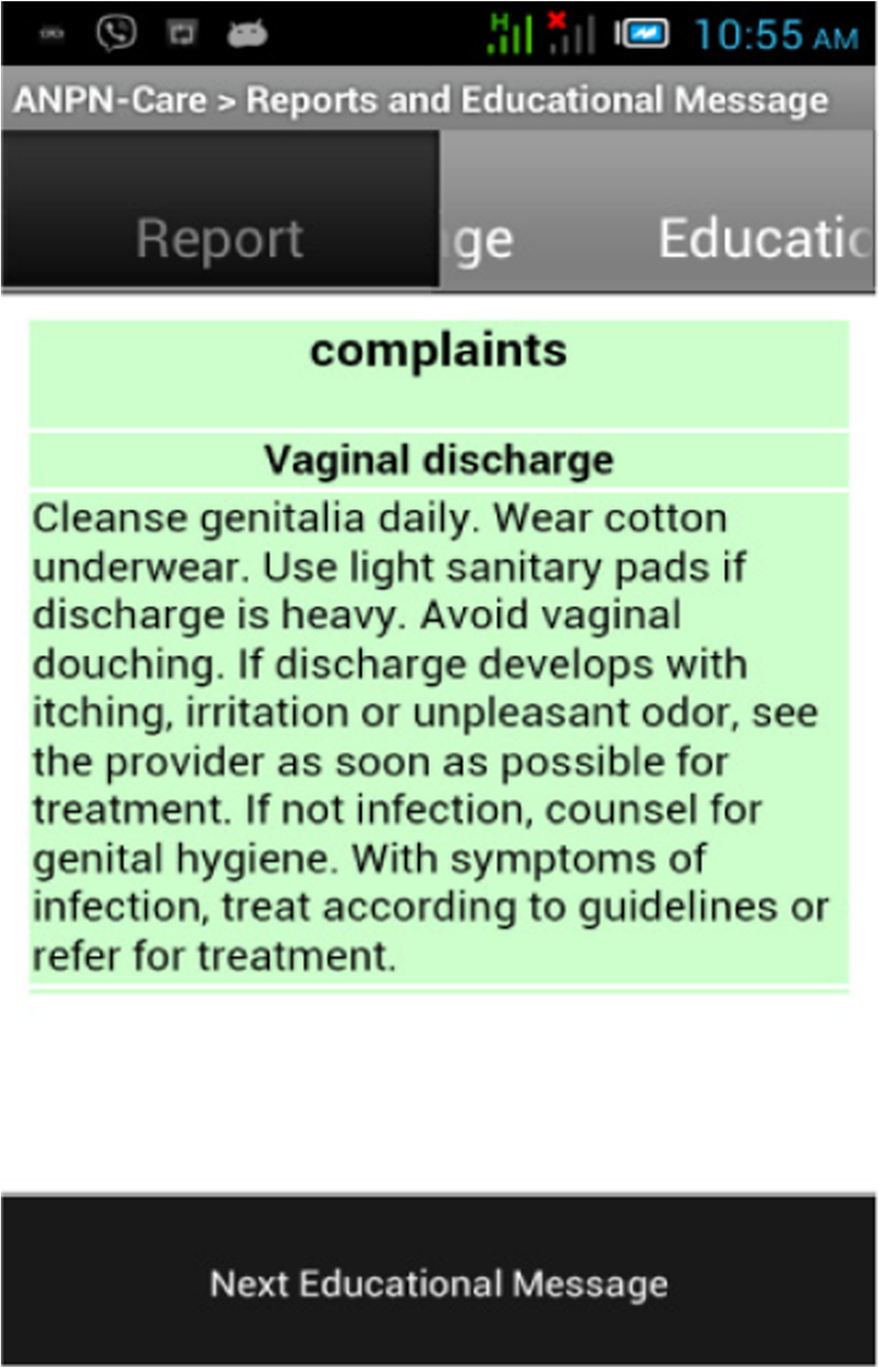
User interface to view educational message

A health worker could also get educational messages from the main server through the front-end mobile application. Educational messages were posted in the back-end system, so that health workers who had access to the front-end mobile application could read the content on their mobile phone (See Fig. 8 for sample educational message on common complaints during pregnancy; namely, vaginal discharge). This feature helped health workers to refresh their knowledge about common complaints during pregnancy and danger signs during pregnancy and delivery.

### Web-based back-end application

**Fig. 9:**
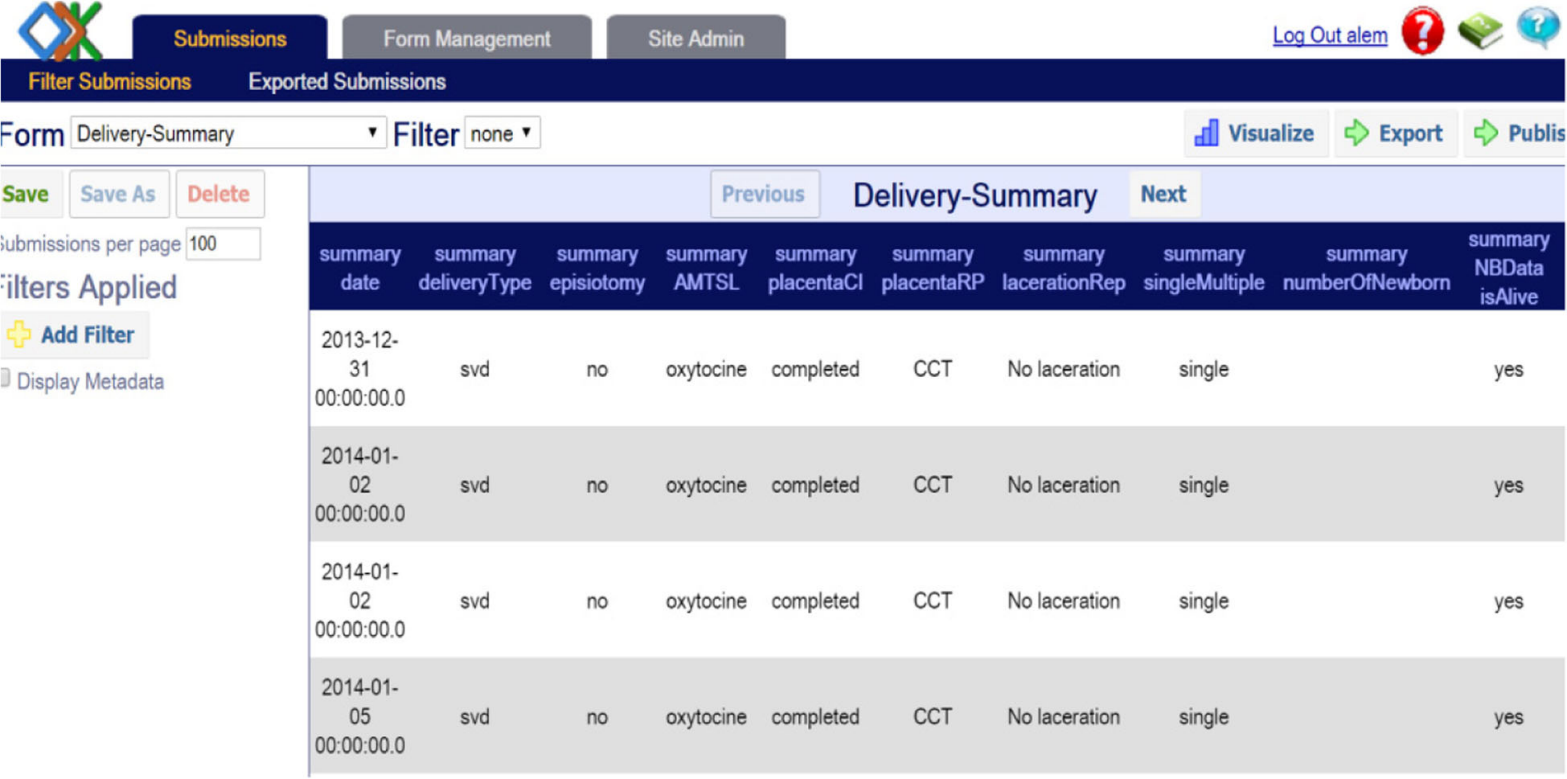
Aggregated mother’s delivery detail when viewed with ODK Aggregate

**Fig. 10:**
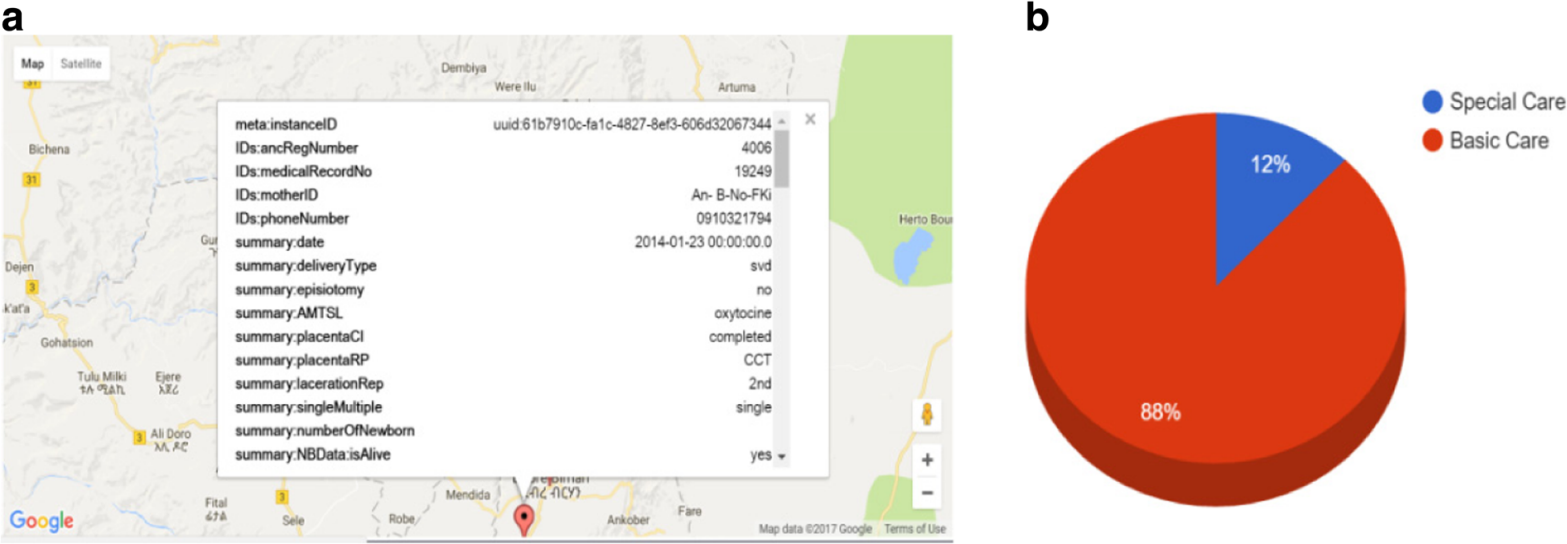
**a** Aggregated data viewed in a map (Map data ©2018 Google), **b** Bar chart to show proportion of mothers that require basic care and specialized care

Back-end users could interact with the system with their personal computer (with internet connectivity) from anywhere. The ODK Aggregate application had a capability to present aggregated content in a tabular and chart format. In addition, for records with GPS coordinates, reported data could be shown in a map. Figure 9 below shows aggregated data of mother’s status during their delivery which can be exported as excel file, while Fig. 10a and b show reported data in a map and chart view respectively.

**Fig. 11:**
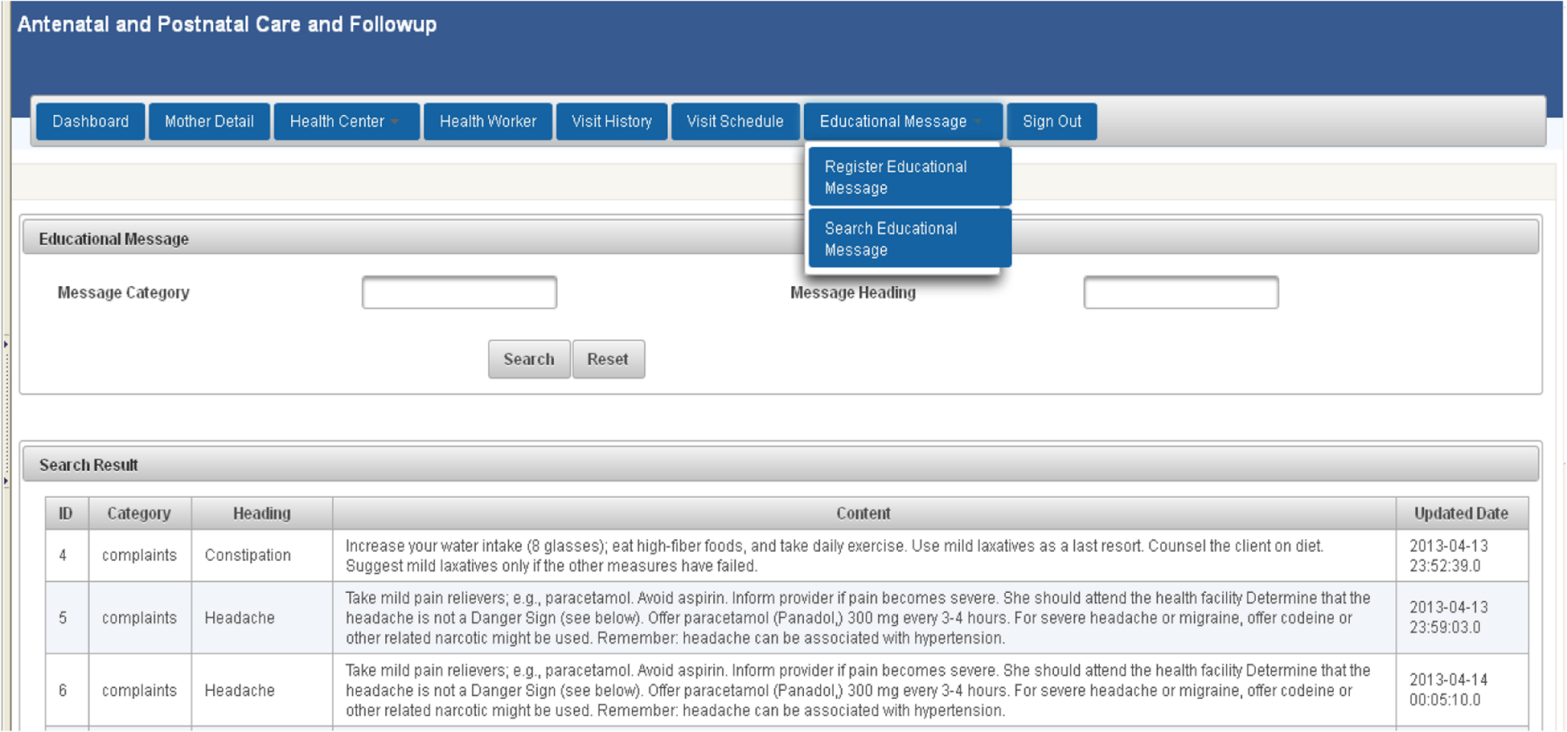
Additional functionalities of the back-end application

To support more back-end functionalities, a separate web-based application was developed and interfaced with ODK Aggregate at the database level. This web-application helped back-end users to view and analyze visit history, visit schedule, and to generate more reports. In addition, users could upload educational messages for health workers from this web-application. Figure 11 shows additional functionalities of the back-end application.

## Implementation experience and lessons learned

### Results

The system was used in 5 health centers since Jan 2014 which was later expanded to an additional 10 centers in June 2016, after training a local IT team at the Zonal office with the expectation of running the system through local resources. Overall, a total of 5927 electronic forms were submitted to the back-end system until June 2016. In this paper, we present the details of each form submission in 2014 for which we have actual client flow data from record reviews. All five health centers used the mHealth system throughout the implementation period, although their monthly ANC client flow varied from 5 to 20. As can be seen in Table 4, the submissions through the mHealth system were slightly lower compared to the actual number of clients who visited those facilities as verified by record reviews.

**Table 4:** Average monthly electronic form submission and actual client flow from record review per health center, 2014

Number of clients	Electronic submission		Client flow from record review
	Mean	SD^b^	
ANC-Classify	14	1	41^a^
ANC-Evaluation	15	1	
ANC-Follow-up	15	0	
Delivery	7	1	13
PNC-Follow-up	8	1	10

^a^ Record for ANC visit per month (not available disaggregated for different forms)

^b^*SD* Standard Deviation

At the time of the write up of this paper, health centers reported they are using part of the applications especially the educational messages and the ability to contact clients who need more frequent follow-up. However, the full range of functionalities of the application (particularly generating reports and providing regular feedback) are not being exploited fully, mainly due to lack of dedicated support staff. The local server set-up for the purpose is also fully functional at the write of this paper.

#### Decision support

##### Correct classification of pregnant women with risk factors

The system classified pregnant women as those who needed specialized care versus those who did not, based on their past and current medical and obstetric history. Accordingly, out of the total 931 ANC classifications made in 2014, the system correctly classified 919 cases (841 Basic Care = and 78 Specialized Care). However, there were 12 cases who were supposed to be classified as needing “Specialized care” and instead classified as needing “Basic care” presumably manually modified by health professionals by mistake as they swipe from page to page on the phone. The basis for defining the classification as correct/incorrect is the presence or absence of various risk factors as recorded by health professionals as indicated in the Ministry of Health protocol [23]. One possible solution for this could be including a ‘warning message’ that pops up whenever someone enters something that is not consistent with previous entries.

##### Calculation of expected date of delivery

The system also had an implemented logic to calculate expected date of delivery (EDD) based on the given last menstrual period (LMP). The following graph shows the pattern of EDD as predicted by the LMP and the actual date of delivery recorded when the woman delivered the child in the health facility.

**Fig. 12:**
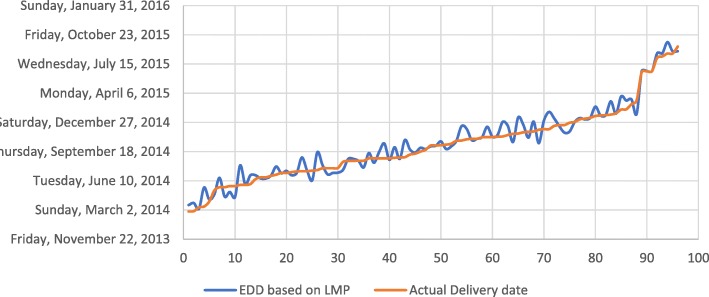
Dates of deliveries as predicted by LMP and actual date of deliveries

The prediction based on LMP and actual date of delivery exactly overlapped in only 7 cases while the prediction was on average earlier than the actual date of delivery by 3 days with a standard deviation of 19 days. See Fig. 12.

#### Timely reporting of data

**Fig. 13:**
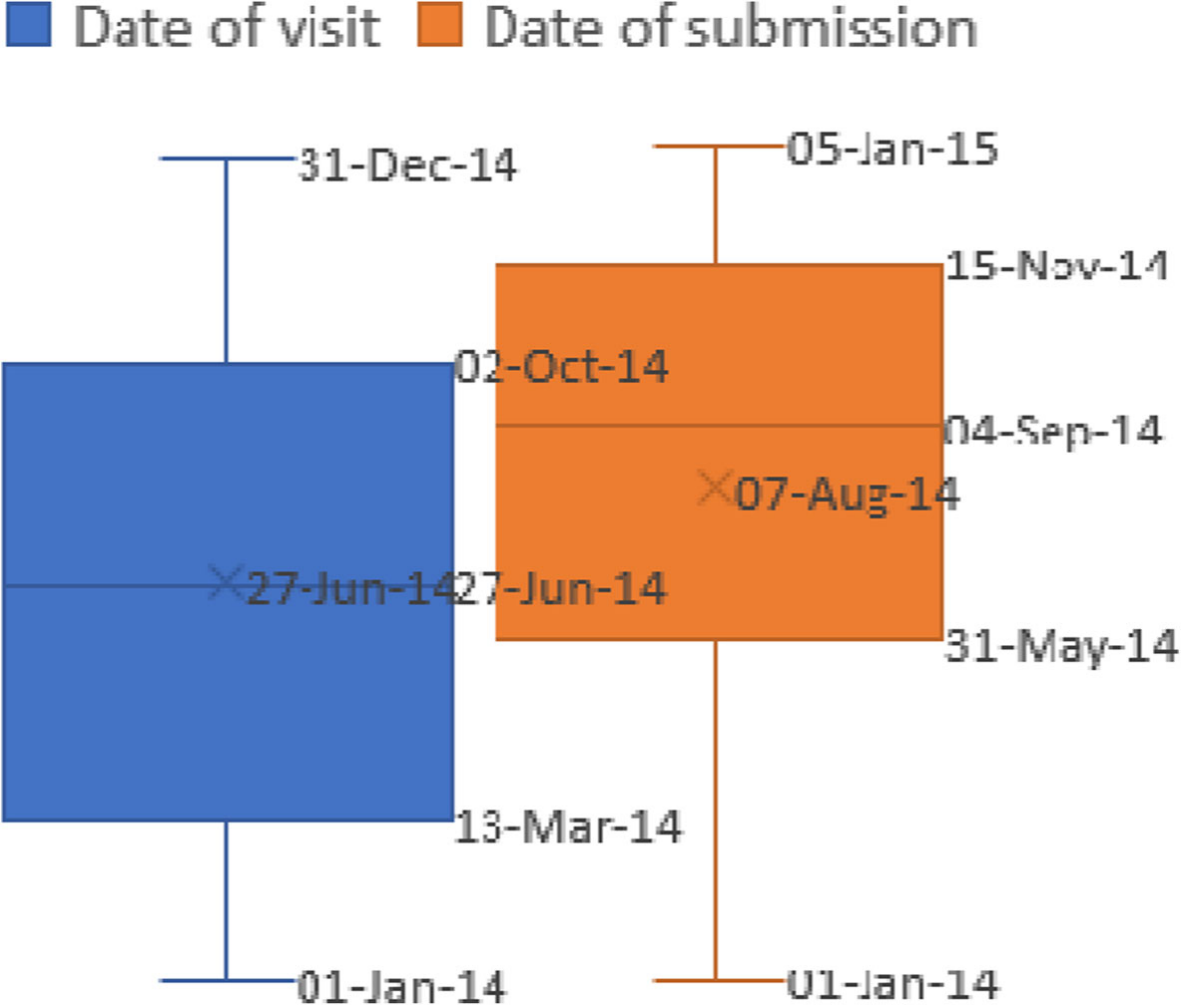
Box and Whisker plot showing the difference between date of client visit and form submission to the server

The application allowed health workers to upload completed forms from their health center or anywhere as long as there was mobile network connectivity. Only 11% of the electronic forms were submitted on the day of the client visit, while an additional 17% of the forms were submitted within 10 days of clients’ visit. On average forms were submitted 39 days after the day of clients visit with a range of 0 to 150 days. We calculated delay in submission of forms as the difference between date of client visit and date the form was uploaded to the server. Figure 13 below visually depicts the delay in electronic form submission. The main reason cited for the delay in submitting forms was substantial workload and poor internet connectivity.

Further, the fact that the system enabled health professionals to easily generate reports on their phone helped them to see patterns and trends in service utilization and avoid the usual tallying they had to do to submit their monthly report to the District health office. For the health workers, this meant saving time needed for manual counting of cases they have seen in every month while it meant improvement in timely aggregation of data and further analysis for local health officials.

#### Lessons learned and discussion

##### Lessons learned

- **mHealth can be developed locally using available resources:** The current mHealth system showed that the technology can be developed using local expertise and resources which made it easier to sustain and adapt to the needs of health professionals without significant delay. The customization process helped build local capacity which was critical for the ongoing support and customization that was required over time. It is for the same reason that the project team continued to provide remote support for other organizations who were interested to expand on the work which has already shown benefits.
- **Open source platform and appropriate technology is essential:** The fact that the current system used open source software, made it possible to expand the service to other health centers, as well as use the application for other purposes including rapid data collection. Two criteria were particularly relevant in choosing appropriate technology among available open source software; namely, the software ability to support relatively complex and long forms and the application’s ability to work offline in an environment where internet data connectivity is unreliable. ODK fulfilled both requirements which was a pre-requisite for the intended intervention to work in primary level facilities.
- **Flexibility to customize the application is key to motivate health workers:** During user training sessions, health workers indicated that they had substantial work load related to reporting their activities monthly and they asked the project team to respond to this request. Health workers believed the flexibility to respond to their immediate needs helped them in adopting the system more quickly than expected. Collecting feedback and continuous improvement also contributed to make the process participatory and create a sense of ownership.

##### Challenges during implementation

Challenges encountered in implementing and sustaining the system had multiple dimensions including issues related to setting standards and service integration, staff turnover, as well as shortcomings related to infrastructure as described below.

- **Lack of unique Identification Number (ID) for ANC clients:** Assigning a unique identifier for pregnant women was a challenge as we needed to follow the woman throughout the period of pregnancy, delivery and postnatal care including those who might shift from one health facility to another. To address this challenge, we generated a unique ID for each woman seeking maternity care services at health facilities which helped to link women having repeat visits. The ID for each woman was generated by combining initials of their name and their address with their medical record number. Lack of unique IDs will likely remain a cross-cutting agenda in Ethiopia until it is fully addressed by the civil registration and vital statistics system [24].
- **Lack of Service integration:** Although service integration is encouraged at all levels of the health care system [19], antenatal care and other maternity care services are usually run in dedicated rooms of health centers by trained professionals. As a result, the current system was designed to work for maternity services only to ensure smooth implementation and documentation of best experiences. Looking forward, there are several constraints which could affect its further development and expansion to other facilities. First, since the system is operating exclusively for maternity services (antenatal; delivery and postnatal care), it is not integrated with other important service components including child health services such as immunization. As a result, health workers were expected to work with the new system as well as with the existing manual system which meant having additional workload. Hence, it will be critical to develop standards for the mHealth ecosystem with service integration and interoperability in mind as those factors could determine feasibility of scale-up in the long-term.
- **Infrastructure related challenges:** Poor network bandwidth and/or electric power interruption at the data center were challenges encountered during implementation although it was infrequent and usually for short periods.
- **Challenges related to phone use and maintenance:** Some of the challenges related to phone use included; inadvertently deleting a form, phone screen becoming insensitive, loss of memory card and occasionally chargers becoming dysfunctional. There was no loss of phones although we replaced some of the phones which became partially dysfunctional after 1 year of implementation.
- **Staff turnover:** The other important challenge was frequent turnover of staff at health facilities as well as support staff in the District/Zonal Health Office, which is also a challenge of the health system in general [25]. It is worth noting, in particular, the loss of one of the key members of the IT and monitoring and evaluation team, which slowed customization and further development of the application in a way that suits the local needs.

## Discussion

Globally, lack of a coherent globally accepted theoretical framework is one of the challenges that is hampering synthesis of the scientific evidence on the effectiveness and efficiency of mHealth interventions particularly in the low-income settings [26–28]. A relatively new framework proposed by Alain B suggests 12 examples of common mHealth applications related to Reproductive, Maternal, Newborn and Child Health [14]. The current mHealth system can be considered as having at least four applications using this framework; namely, data collection/reporting, electronic health records, electronic decision support, and provider education along the continuum of care including antenatal, delivery and postnatal care.

The present mHealth system showcases how a successful and sustainable mHealth intervention can be designed and implemented in a low-income setting, using the available human and technological resources. The key elements for success of implementation and sustainability, in our view, were using an open source platform and focusing on locally relevant priority conditions in consultation with local officials. To this end, the project made several improvements to respond to the needs in the local environment as illustrated by the following examples. First, recognizing the significant burden of reporting of health workers, we developed a reporting feature. This allowed health workers to see the number of clients they examined for any given period on their phones. This feature was developed at the request of health workers after the project was launched. Second, we developed a web-based application that was linked with the main ODK aggregate server that used the data to generate ready-made reports, showing performance of health workers or health facilities for any given period. This feature was developed mainly for local health officials. Finally, it was easier to accommodate evolving demands as the project was rolled out, including using the platform to conduct surveys using a customized version of ODK which supported the local language (Amharic) and local (Ethiopian) calendar.

Further, the system allowed health officials to observe and analyze data patterns that would have been impossible using the routine reporting system which was limited to few indicators. Examples include getting information on the percentage of clients who had undergone certain necessary procedures such as episiotomy and what proportion had third-degree tear, which helped to identify areas where additional training and service improvement might be needed.

A review of impact of mHealth projects in various areas of community health in Africa by Eva et al. indicate that the most successful interventions are the ones least affected by contextual challenges because of their simple design and modest objectives or those which involved rare efforts to address circumstantial challenges [16]. Important challenges reported in the mHealth literature include lack of user-centered application, conflicting health system priorities and insufficient local government commitment and support [11, 27, 29]. The current intervention tried to address these challenges through early engagement of local public officials and focusing the intervention on their priority problems. Finally, we acknowledge that the project could have benefited from a well-designed user survey as an input to improve the user-friendliness of the application and designing the forms in a way that minimizes errors.

## Conclusions

In summary, this paper provides detailed description of the steps involved in designing and implementing an open source mHealth platform and its potential use in day-to-day activities of health workers and officials which could help to replicate similar approaches in the future.

Based on the experience from the current implementation, the authors believe that responding to pressing priority problems at the lowest level of the health care system is key for a successful operation of mHealth interventions although keeping standards and uniformity across the health system is also an important consideration.

## Abbreviations

ANC: Antenatal care
ID: Identification Number
ODK: Open Data Kit
PNC: Postnatal Care
